# Anticancer Drug-Incorporated Layered Double Hydroxide Nanohybrids and Their Enhanced Anticancer Therapeutic Efficacy in Combination Cancer Treatment

**DOI:** 10.1155/2014/193401

**Published:** 2014-04-17

**Authors:** Tae-Hyun Kim, Gyeong Jin Lee, Joo-Hee Kang, Hyoung-Jun Kim, Tae-il Kim, Jae-Min Oh

**Affiliations:** ^1^Department of Chemistry & Medical Chemistry, College of Science & Technology, Yonsei University, Wonju, Gangwon-do 220-710, Republic of Korea; ^2^Department of Biosystems and Biomaterials Science and Engineering, College of Agriculture and Life Sciences, Seoul National University, Seoul 151-921, Republic of Korea; ^3^Ewha-Solvay Research & Innovation Center, 150 Bukahyun-ro, Seodaemun-gu, Seoul 120-140, Republic of Korea

## Abstract

*Objective*. Layered double hydroxide (LDH) nanoparticles have been studied as cellular delivery carriers for anionic anticancer agents. As MTX and 5-FU are clinically utilized anticancer drugs in combination therapy, we aimed to enhance the therapeutic performance with the help of LDH nanoparticles. *Method*. Anticancer drugs, MTX and 5-FU, and their combination, were incorporated into LDH by reconstruction method. Simply, LDHs were thermally pretreated at 400°C, and then reacted with drug solution to simultaneously form drug-incorporated LDH. Thus prepared MTX/LDH (ML), 5-FU/LDH (FL), and (MTX + 5-FU)/LDH (MFL) nanohybrids were characterized by X-ray diffractometer, scanning electron microscopy, infrared spectroscopy, thermal analysis, zeta potential measurement, dynamic light scattering, and so forth. The nanohybrids were administrated to the human cervical adenocarcinoma, HeLa cells, in concentration-dependent manner, comparing with drug itself to verify the enhanced therapeutic efficacy. *Conclusion*. All the nanohybrids successfully accommodated intended drug molecules in their house-of-card-like structures during reconstruction reaction. It was found that the anticancer efficacy of MFL nanohybrid was higher than other nanohybrids, free drugs, or their mixtures, which means the multidrug-incorporated LDH nanohybrids could be potential drug delivery carriers for efficient cancer treatment via combination therapy.

## 1. Introduction

Recently, nanotechnology-based drug delivery systems have emerged as powerful methods not only to enhance drug efficacy but also to minimize side effects of cancer chemotherapy [1–6]. Many kinds of engineered nanomaterials such as liposomes [7, 8], polymeric nanoparticles [9–11], porous nanomaterials [12–14], and 2-dimensional layered nanomaterials [4, 15–18] have been investigated to safely preserve, to release in controlled manner, and to efficiently deliver anticancer agents. Among them, 2-dimensional layered nanomaterials, such as layered double hydroxide (LDH), have attracted interests as potential cellular delivery nanocarriers for anionic anticancer drugs including methotrexate (MTX) and 5-fluorouracil (5-FU) which are generally known to have low affinity to the negatively charged cell membrane.

LDHs having general chemical formula, [M(II)_1-*x*_M(III)_*x*_ (OH)_2_]^+^(A^*n*−^)·*m*H_2_O (M(II): divalent metal, M(III): trivalent metal, A: interlayer guest anion, 0 < *x* < 1; *m* and *n* are integers), are unique synthetic minerals having positively charged layers [19]. The interlayer anions are stabilized between the LDH layers through electrostatic interaction and safely protected from external harsh conditions [1, 17, 20]. The anionic chemical species can be intentionally incorporated into LDHs through various ways: coprecipitation, ion-exchange reaction, reconstruction, and exfoliation-reassembly [21, 22]. In the coprecipitation, solution containing metal cations and anionic species is titrated with alkaline solution to* in situ *form anion-incorporated LDH. Ion-exchange reaction can be carried out with the LDHs having easily exchangeable anions (NO_3_^−^ or Cl^−^), which are exchanged with intended anions upon concentration gradient. For reconstruction, LDHs are pretreated in mild temperature to produce mixed metal oxide, M(II)_1−*x*_M(III)_*x*_O_(2+*x*)/2_, which then recovers layered LDH structure upon addition of water and intended anions. In exfoliation-reassembly route, LDH is treated with an appropriate solvent (usually formamide) to be delaminated into single sheets, which can be restacked to the LDH structure in the presence of anionic species.

There have been intensive studies, during the last decades, on the development and optimization of LDHs as drug nanocarriers to enhance cellular uptake, efficacy, and target-specificity of anticancer drugs [23–25]. Anionic anticancer drug, MTX, which is clinically utilized in the treatment of osteosarcoma or breast cancer [26, 27], was introduced to MgAl- or ZnAl-LDH through coprecipitation and ion-exchange reactions [1, 28]. It has been reported that the MTX/LDH nanohybrid can be efficiently internalized by cancer cell via clathrin-mediated endocytosis which is different from the general uptake pathway of drug molecule itself, resulting in high anticancer efficacy [23, 29]. Furthermore, the MTX/LDH nanohybrids were reported to overcome drug resistance in cancer cells which is a major problem of clinical chemotherapy [30], as well enhancing their cellular recognition through surface modification with cancer cell targeting ligand, folic acid [24]. Similarly, anionic drug, 5-FU, was reported to form stable drug/LDH nanohybrid by coprecipitation [25, 31]. Compared with 5-FU drug only, the 5-FU/LDH nanohybrid not only enhanced anticancer activity* in vitro* cell line but also increased systemic circulation* in vivo *[25].

Generally, MTX and 5-FU are clinically utilized in combination for the efficient treatment of cancer, which is generally known as CMF (cyclophosphamide, methotrexate, and 5-fluorouracil) therapy, to reduce drug resistance, to supplement each other, and to achieve additional synergism. It has been reported that the combinatorial treatment of MTX and 5-FU can positively affect the anticancer mechanism of each other by adjusting the administration sequence [32, 33]. Inspired by the combination therapy concept, we tried to apply LDH nanocarrier to the codelivery of MTX and 5-FU. In spite of intensive researches on drug delivery nanocarriers, there have been only a few reports on the drug delivery concept applied to the combination therapy such as organic carriers like liposomes, polymeric particles, or lipid capsules [34–37]. However, there has been no report on the utilization of inorganic nanocarriers like LDH in combination therapy, as far as we know.

In this study, we prepared three kinds of drug/LDH nanohybrids, each containing MTX, 5-FU, and their combination, respectively. The homogeneous control of particle size and morphology in drug/carrier system is known to strongly affect the delivery efficacy [23, 29]. In order to control those properties, we adapted reconstruction method, which has not been widely utilized in preparing drug/LDH nanohybrids. The precursor of reconstruction, CO_3_-LDH, is well known to have controlled size and morphology compared with NO_3_-LDH, a precursor of ion-exchange. The reconstruction starting from the CO_3_-LDH having well controlled size and morphology would result in the fine-control of drug/LDH nanohybrids. We are going to demonstrate the physicochemical properties such as particle size, morphology, and surface charge of prepared nanohybrids considering the biological behavior of LDH nanoparticles. And the enhanced drug efficacy and potential of LDH system as combination therapy will be described with* in vitro* cell line test utilizing HeLa human cervical adenocarcinoma cells.

## 2. Materials and Methods

### 2.1. Synthesis of Drug/LDH Nanohybrids

Pristine LDH with chemical formula, Mg_2_Al (OH)_6_(CO_3_)_0.5_·*m*H_2_O, having homogeneous particle size distribution was prepared according to the previous report [38]. Typically, base solution (0.75 M of NaOH and NaHCO_3_) was added dropwise to the mixed metal solution (0.1875 M of Mg^2+^ and 0.0973 M of Al^3+^) until pH reached ~9.5 under vigorous stirring. Then the white suspension was aged under hydrothermal condition at 150°C for 48 hours. The final product was collected by centrifugation and was thoroughly washed with deionized water and then dried with freeze-dryer.

In order to incorporate drug molecules into LDH, we utilized reconstruction route in which thermally treated LDH recovered its structure under the existence of water and intended anionic molecules. First, pristine LDH was calcined at 400°C for 8 hours to obtain mixed metal oxide, Mg_2_AlO_7/2_. Drug solutions were prepared as follows: MTX and 5-FU powder were suspended in water, respectively, and 0.3 M NaOH and NH_4_OH were added until pH reached approximately 8 to solubilize both drug molecules. Calcined LDH powder (50 mg) was suspended into either MTX (0.0057 M) or 5-FU (0.0114 M) solution (90 mL) and vigorously stirred under N_2_ atmosphere for 24 hours to obtain ML (MTX/LDH) or FL (5-FU/LDH) nanohybrids, respectively. In order to introduce MTX and 5-FU into the LDH lattice simultaneously (MFL), (MTX + 5-FU) mixed solution was prepared by adding minimal amount of NH_4_OH. Then, 50 mg of calcined LDH powder was suspended into the mixed drug solution (0.0021 M (MTX) and 0.032 M (5-FU)) and vigorously stirred under N_2_ atmosphere for 48 hours. All the nanohybrids were collected by centrifugation and washed with decarbonated water. The prepared nanohybrids were stored in slurry state just after centrifugation at 12,000 rpm and diluted with saline for biological assay. A part of slurry was dried in vacuum for physicochemical characterizations.

### 2.2. Characterization

The particle size and morphology of pristine LDH, calcined LDH, and drug/LDH nanohybrids were investigated with scanning electron microscopy (SEM) on Quanta 250 FEG. For SEM measurement, all the samples were diluted with deionized water to the concentration ~0.4 mg/mL and vortexed for dispersion. Then a drop of suspension was put on the silicon wafer which was previously cleaned with piranha solution. After drying water in vacuum, the surface of sample was coated with Pt/Pd plasma for 50 seconds and images were collected by 30 kV of accelerated electron beam. In order to obtain average particle size of samples and to analyze statistically, randomly selected 160 particles from at least 6 different spots were utilized.

The powder X-ray diffraction (XRD) patterns of all the samples were obtained by Bruker AXS D2 Phaser (LYNXEYE detector) using Ni-filtrated Cu-K_*α*_ radiation (*λ* = 1.5406 Å) with 1 mm of air-scattering slit and 0.1 mm of equatorial slit. For the XRD measurement of drug/LDH nanohybrids, slurry samples were spread on flat slide glass and dried at room temperature. XRD patterns were collected with degree step of 0.02° and time step increments of 0.5 sec/step from 3 to 30°.

Fourier transformed infrared (FT-IR; Perkin Elmer, Spectrum one B.v5.0) was performed with standard KBr methods. FT-IR spectra were recorded from 450 to 2000 cm^−1^ with resolution 8 cm^−1^ and 16 times repetition.

### 2.3. Drug Content Quantification

In order to determine the chemical formulae of drug/LDH nanohybrids and to quantify the amount of drugs in the nanohybrids suspension for biological assay, thermal analysis, elemental analysis, and liquid chromatography were carried out. Thermogravimetric analysis was performed with SDT Q600 (TA instruments), in the temperature range from 23 to 1000°C with heating rate 10°C/min under 100 mL/min of air flow. In the CHNS elemental analysis utilizing Perkin Elmer, PE 2400, acetanilide and empty tin capsule were used for blank correction (error rate below 0.05%). All the samples were subjected to calcination at 1000°C with He, N_2_, and O_2_ mixture gas flow for 18 seconds. The drug contents (MTX and 5-FU) in both powder and suspension were quantified with high performance liquid chromatography (HPLC; Younglin, YL9100 HPLC) with C18 column (ZOBAX Eclipse, 4.6 × 150 mm, Agilent). Before HPLC measurement, samples were treated with phosphate buffer solution (pH ~2) by stirring for 10 min and sonicating for 10 min, in order to dissolve LDH lattices. For MTX analysis, mobile phase of 0.05 M KH_2_PO_4_ and 10% acetonitrile was utilized with flow rate 1.0 mL/min, column temperature of 40°C, and UV absorption detector at 304 nm [30]. For 5-FU analysis, mobile phase of 1% acetonitrile in deionized water was utilized with flow rate 1.0 mL/min, column temperature of 25°C, and UV adsorption detecting at 246 nm [25].

### 2.4. Colloidal Property Evaluation

The surface charge of drug/LDH nanohybrids was measured by *ζ*-potentiometer (Otsuka electronics, ELS-Z1000) and the zeta potential value was calculated by Smoluchowski equation with the program provided by Otsuka. The hydrodynamic size of drug/LDH nanohybrids was determined by dynamic light scattering method with Marquardt analysis (Otsuka electronics, ELS-Z1000). For the measurement of both zeta potential and hydrodynamic size, drug/LDH nanohybrids were diluted to concentration ~0.2 mg/mL with deionized water, Dulbecco's modified eagle medium (DMEM), and DMEM with 10% of fetal bovine serum (FBS), respectively.

### 2.5. Biological Assay

The anticancer activity of drug/LDH nanohybrids was investigated by MTT (3-(4,5-dimethylthiazol-2-yl)-2,5-diphenyltetrazolium bromide) assay, based on drug concentration and LDH (inorganic part) concentration, respectively. HeLa cells were seeded in 96-well plates in 100 *μ*L DMEM (10% FBS) at a density of 1 × 10^4^ cells/well. Having achieved 70–80% confluency after 24 hours, the cells were exposed to 100 *μ*L solutions (DMEM with 10% FBS) containing free drugs or drug/LDH nanohybrids. MTX + 5-FU mixture and ML + FL mixture were also used to compare their cancer cell killing efficacies with MFL nanohybrid, respectively. Their mixing ratios were determined by drug content (wt%) in MFL nanohybrid (MTX : 5-FU = 1 : 1.10). The cells were incubated for 2 days, followed by treatment with 25 *μ*L of MTT stock solution (2 mg/mL DPBS) and further incubated for 2 hours at 37°C. After removing each medium carefully, 150 *μ*L of DMSO was added to each well to dissolve the formazan crystals formed by proliferating cells. The absorbance of formazan solution was measured at 570 nm using a microplate reader (Synergy H1, Biotek, USA). Results were presented as relative cell viabilities (RCV, percentage values relative to values of untreated control cells). All experiments were performed in triplicate.

## 3. Results and Discussion

**Figure 1:**
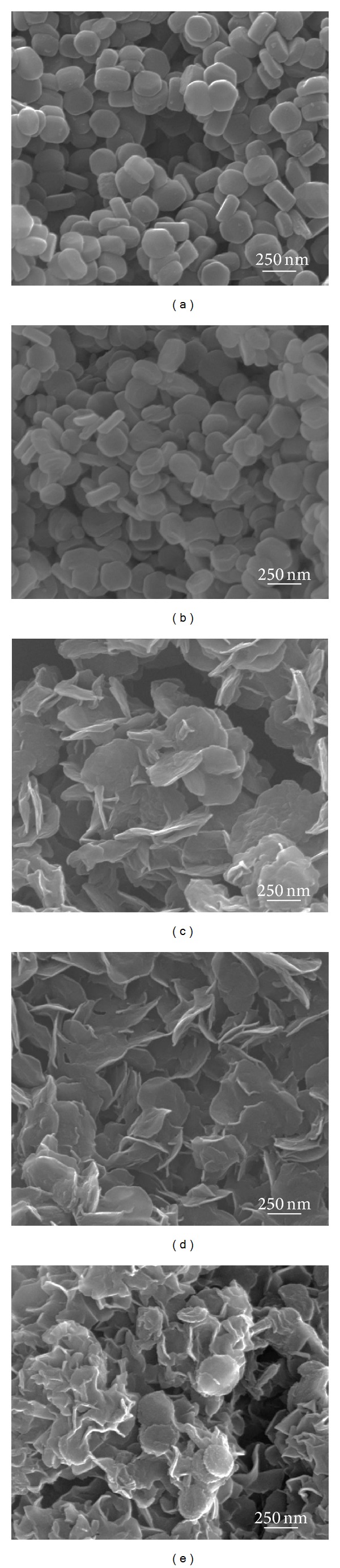
Scanning electron microscopic images of (a) MgAl-LDH, (b) mixed metal oxide, and drug/LDH nanohybrids: (c) ML, (d) FL, and (e) MFL.

Size and morphology of pristine Mg_2_Al(OH)_6_(CO_3_)_0.5_-LDH, calcined LDH, and drug/LDH nanohybrids were visualized with SEM as displayed in Figure 1. The particle size distribution of each sample was highly homogenous and the morphology showed characteristic feature of LDHs and organic-incorporated LDHs as reported previously [38, 39]. The lateral particle size and thickness of pristine LDH were ~233 ± 12 and ~100 ± 16 nm, respectively. Those values for calcined LDHs were determined to be ~236 ± 13 and ~73 ± 11 nm, respectively. From Student's* t*-test with 95% confidentiality, it was confirmed that the lateral particle size of LDH preserved after calcinations while the particle thickness reduced significantly. It has been reported that the LDH layers topochemically underwent dehydration, dehydroxylation, and decarbonation during heat treatment [40]. Therefore, the* xy*-plane was not altered much and the interlayer distance reduced significantly with the removal of interlayer carbonate. The morphology of drug/LDH nanohybrids was fairly different from that of LDH or calcined one, showing rosette-like shape with very much reduced particle thickness, less than 20 nm, and bending of layers. Decrease in thickness was attributed to the partial delamination of LDHs during intercalation of drug molecules like MTX or 5-FU. The rosette-like structure or house-of-cards structure, which is due to the enhanced particle-edge interaction, has been reported as a typical feature of organic/LDH hybrids obtained by reconstruction route [39, 41]. Although it was difficult to estimate the lateral particle size of drug/LDH nanohybrids due to the randomly stacked thin and bended layers, we found that the lateral sizes of drug/LDH nanohybrids were ~225 ± 14, ~236 ± 12, and ~234 ± 14 nm for ML, FL, and MFL, respectively, from repeated SEM measurements (see Figure S1 in Supplementary Material available online at http://dx.doi.org/10.1155/2014/193401). From the results that the lateral particle sizes were preserved during reconstruction synthesis and only thickness slightly changed, we could suggest reconstruction as an appropriate way to topochemically incorporate drug molecules into LDHs preserving the 2-dimensional structure of LDH nanocarrier.

**Figure 2:**
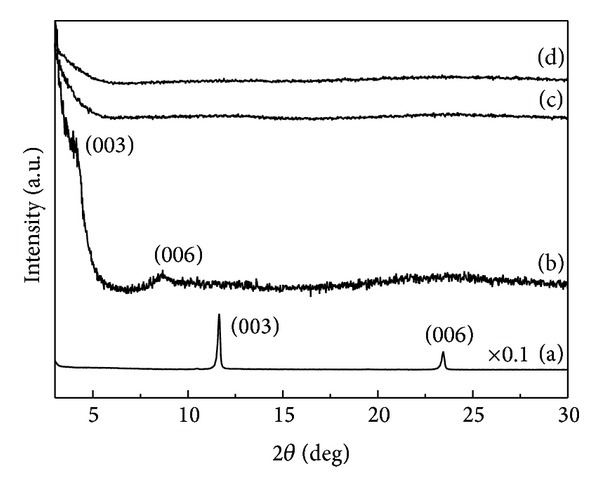
Powder X-ray diffraction patterns of (a) pristine MgAl-LDH and nanohybrids, (b) ML, (c) FL, and (d) MFL.

We checked the crystal structure of LDHs during incorporation of drug molecules with powder X-ray diffraction pattern (Figure 2). Pristine LDH showed typical diffraction pattern of hydrotalcite (JCPDS number 14-0191) with hexagonal crystal lattice [19]. The (*003*) peak at around 2-theta 11.66° corresponding to the* d*-spacing of 0.76 nm was typical peak of carbonate intercalated LDHs. The sharp peak stood for the well-developed layered stacking along* c*-axis. ML nanohybrid showed (*003*) and (*006*) peaks at 4.08° and 8.66°, respectively, which corresponded to the interlayer distance of 1.68 nm. Taking into account the molecular dimension of MTX, it was considered that the MTX molecules were arranged in the interlayer space with tilting angle of ~38° towards* c*-axis of LDH's layer stacking, which was in good agreement with the previous report [1]. Both FL and MFL nanohybrids showed amorphous-like XRD pattern in the 2-theta range of 3~30° without showing (*00l*) peaks. The particle thickness of those nanohybrids was determined to be fairly thin (<20 nm) from the SEM (Figure 1) and furthermore the particles were randomly stacked forming house-of-cards structure. As the X-ray diffraction detected well-ordered crystalline (*hkl*) planes with long-range ordering, it was not easy to observe (*00l*) peaks in the drug/LDH nanohybrids having thin particle thickness and random orientation. The relatively low (*003*) intensity in the ML nanohybrids compared with pristine one was also explained by the random stacking of thin particles.

**Figure 3:**
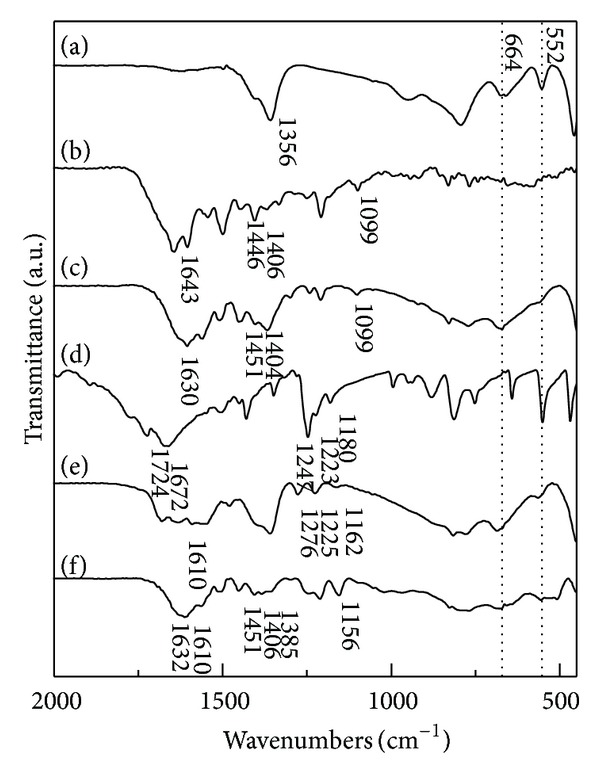
Fourier transformed infrared spectra of (a) MgAl-LDH, (b) methotrexate, (c) ML, (d) 5-FU, (e) FL, and (f) MFL.

The incorporation of drug molecules into LDH layers in drug/LDH nanohybrids was successfully confirmed with FT-IR (Figure 3). The infrared spectrum of MTX molecules (Figure 3(b)) showed characteristic bands of MTX molecules at 1643, 1446, 1099, and 1406 cm^−1^, which were attributed to the stretching vibration of COOH, C=C, and C–N in the aromatic backbone and bending vibration of –OH [30]. We could observe the typical IR modes of MTX backbone such as C=C stretching vibration (1453 cm^−1^) and C–N stretching vibration (1099 cm^−1^) in the ML nanohybrids (Figure 3(c)). It was worthy to note that the COOH stretching vibration of MTX (1643 cm^−1^ in Figure 3(b)) disappeared in the IR spectrum of ML nanohybrid, and instead asymmetric (*ν*_as_) and symmetric stretching vibration (**ν**_*s*_) of COO^−^ appeared at 1630 and 1404 cm^−1^, respectively. This result suggested that the MTX molecules were stabilized through electrostatic interaction with positively charged LDH layer. The difference between *ν*_as_ and **ν**_*s*_ (Δ_as−*s*_) is generally utilized to evaluate the state of carboxylate as well as the degree of interaction between carboxylate and cationic charges. According to the previous report, the Δ_as−*s*_ value of 226 cm^−1^ in this study well explained the electrostatic stabilization of MTX in LDH interlayer space [30]. Although we could not find the highly ordered (*00l*) peaks from the XRD (Figure 2), we could confirm that the MTX molecules were well incorporated into LDHs from IR results.

A similar feature was also observed in the spectra of 5-FU and FL nanohybrids. The IR spectra of 5-FU exhibited characteristic peaks at 1724, 1672, 1247, 1223, and 1180 cm^−1^ corresponding to the vibration stretching of C=O in imide, amide (I) C=O stretching vibration, amide (III) C=O (overlapping with N–H in-plane bend and C–N stretching), *ν*_CF_, and *δ*_CH_, respectively [31, 42]. In the IR spectra of FL, we could find the 5-FU's characteristic peaks attributed to the C=O (1276 cm^−1^), *ν*_CF_ (1225 cm^−1^), and *δ*_CH_ (1162 cm^−1^), respectively. After hybridization, the peak corresponding to C=O in imide (1724 cm^−1^) disappeared and a broad band in the region of 1500–1680 cm^−1^ attributed to the overlapping of C=C, C=N, and C=O stretching vibration appeared, which was in good agreement with the previous report on 5-FU-incorporated LDH [31]. Furthermore, we could clearly observe a newly developed peak at 1610 cm^−1^ which indicated the strong interaction between 5-FU molecules and cationic substance [42]. Thus the FL nanohybrid was considered to contain the 5-FU moiety through electrostatic interaction.

In the case of MFL nanohybrid, all characteristic peaks of MTX and 5-FU backbone as well as peaks indicating the electrostatic interaction between anionic drug molecules (MTX and 5-FU) and cationic LDH layers were clearly observed in IR spectrum. Therefore, we could conclude that all the three nanohybrids well incorporated intended drug molecules through electrostatic interaction, and the co-incorporation of two different drug molecules through reconstruction route was successful.

In order to evaluate cellular drug delivery efficiency utilizing nanocarrier, it is of importance to precisely determine the drug loading capacity and content in the drug/carrier system. We utilized various analytical methods to precisely determine the drug contents in drug/LDH nanohybrids. As LDH generally contained water molecules to some extent as crystal water which evaporated in the temperature range 100–250°C, we could determine the water content in the nanohybrids from TG analyses. Then the drug content was assessed with two different methods, CHNS elemental and HPLC analyses, each confirming the other's result. We hypothesized that the interlayer space of nanohybrids is filled with both anionic drug molecules and hydroxyl anions to balance the charge with LDH layers. As the reconstruction reaction proceeded in decarbonated water under N_2_ blanket, there only existed two types of anions, drug molecules and hydroxides, in the reaction vessel. The chemical formulae and drug content are summarized in Table 1. The drug content of ML and FL was determined to be 28.3 and 10.9 wt%, respectively. Although those values were less than the maximum possible drug content for drug/LDH nanohybrid (Table 1), the values were still practical compared with other nanodrug delivery systems such as liposomes (~10 wt% of drug content) [43]. Furthermore, the relatively low drug content was not considered as a critical disadvantage, taking into account that the drug/LDH nanohybrids were reported to transport massive amount of drugs [2]. The drug content of MFL, the drug co-incorporated nanohybrid, was 19.0 and 21.0 wt% for MTX and 5-FU, respectively, of which total content is sufficiently high (~40 wt%), suggesting the potential of reconstruction as a preparative method in combination therapeutic delivery system. The relatively high drug content in the MFL compared with ML or FL might be attributed to the *π*-*π* interaction between the aromatic rings in MTX and 5-FU making best use of the interlayer space.

**Figure 4:**
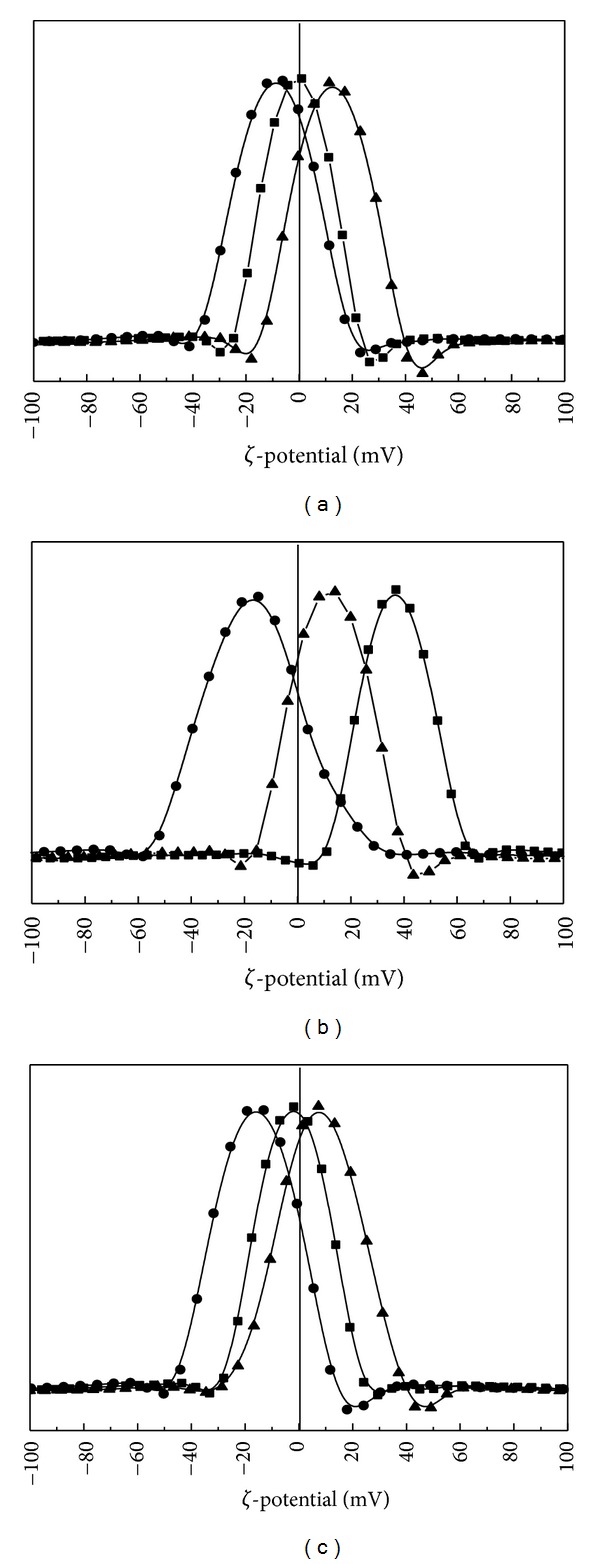
(a) The surface charge (*ζ*-potential) distribution graph of ML in deionized water (—■—) at pH 8.3 (average *ζ*-potential: −0.61 mV), DMEM (—●—) at pH 7.88 (average *ζ*-potential: −8.94 mV), and DMEM with 10% FBS (—▲—) at pH 7.86 (average *ζ*-potential: 12.47 mV). (b) The surface charge (*ζ*-potential) distribution graph of FL in deionized water (—■—) at pH 8.35 (average *ζ*-potential: 36.99 mV), DMEM (—●—) at pH 7.95 (average *ζ*-potential: −17.07 mV), and DMEM with 10% FBS (—▲—) at pH 8.02 (average *ζ*-potential: 12.19 mV). (c) The surface charge (*ζ*-potential) distribution graph of MFL in deionized water (—■—) at pH 8.47 (average *ζ*-potential: −2.08 mV), DMEM (—●—) at pH 8.02 (average *ζ*-potential: −15.96 mV), and DMEM with 10% FBS (—▲—) at pH 8.03 (average *ζ*-potential: 7.64 mV). The vertical line stands for the zeta potential value of 0 mV.

Most of the nanodrug delivery systems for cellular delivery are administered in aqueous suspension or solution state. Thus it is essential to investigate their physicochemical properties in suspension, such as zeta potential and hydrodynamic size. Especially, zeta potential which reflects the surface charge is considered as an important factor to decide interaction between nanomaterials and cells [44–46]. Usually plasma membranes of mammalian cells are negatively charged due to the phospholipid bilayers and carbohydrates embedded in the membrane [47, 48]. Many reports highlighted that the positively charged nanomaterials interact more actively with cell membranes [49–53], eventually resulting in the enhanced cellular uptake. LDHs are usually known to have positive layer charge; however, the surface charge of LDH can be affected by both the surface coated molecules and the type or concentration of electrolytes or solutes in the suspending media [54]. Therefore, we carried out zeta potential measurement in three different media including deionized water and cell culture medium (DMEM) with or without FBS. The zeta potential values of three nanohybrids in deionized water were −0.61, +36.9, and −2.08 mV for ML, FL, and MFL, respectively. According to the zeta potential distribution graph, more than half of the zeta potential values lie in the positive region (Figure 4). Nanohybrids having MTX moiety (ML and MFL) showed relatively negative surface charge compared with FL. As MTX had two anionic carboxylate groups and some of the MTX molecules are attached on the outer surface of LDH, it was thought that the surface charge of those nanohybrids (ML and MFL) showed negative values.

When the nanohybrids were suspended in the DMEM containing many anionic electrolytes, the values significantly shifted to the negative region, showing −8.94, −17.07, and −15.96 mV for ML, FL, and MFL nanohybrids, respectively. It has been well reported that the zeta potential of nanomaterials changes according to the adsorption of countercharged chemical species [53, 55, 56]. However, it is worthy to note that still a part of zeta potential graph lies in the positive region. Upon FBS addition, all the zeta potential values shifted again to the positive direction, exhibiting +12.47, +12.19, and +7.64 mV for ML, FL, and MFL nanohybrids, respectively. It was considered that the albumin proteins in the FBS having both negative and positive charge can neutralize the surface charge to recover original positive surface charge of LDHs. It has been also reported in the previous researches that the addition of proteins recovers (or shifts zeta potential to the positive direction) the surface charge of nanomaterials [57, 58].

**Figure 5:**
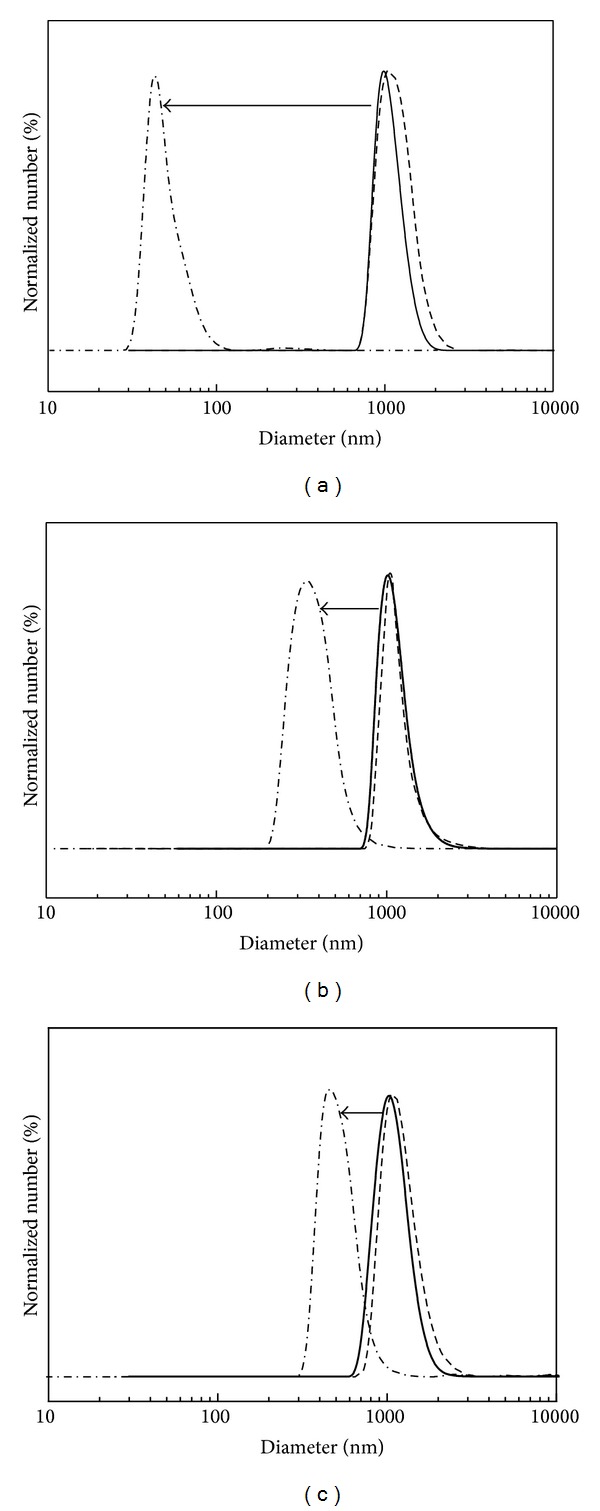
Hydrodynamic size distribution of nanohybrids (a) ML, (b) FL, and (c) MFL, dispersed into deionized water (solid line), DMEM (dashed line), and DMEM with 10% FBS (dashed dot line).

The hydrodynamic size distributions of nanohybrids in various suspending media are displayed in Figure 5. All the nanohybrids in deionized water and DMEM media showed agglomeration with average hydrodynamic size values of ~980, ~1010, and ~1021 nm in deionized water and ~1070, ~1050, and ~1070 nm in DMEM for ML, FL, and MFL, respectively. And the size distribution lies in the region of 600–2000, 700–2000, and 600–2000 nm in both deionized water and DMEM. The organic/LDH nanohybrids usually showed formation of agglomerate due to the strong interparticle interaction [59, 60]. However, it did not mean that the primary particles of nanohybrids with ~200 nm lateral size strongly aggregated in the aqueous media, as they can be easily stabilized into small assembly of particles under appropriate treatment of stabilizing agents. Nanomaterials produced in powder which form agglomerates can be separated into single particles in the presence of stabilizer such as surfactants or proteins [61]. In this study, we also observed that the degree of agglomeration strongly reduced with the addition of FBS containing albumin protein. The average hydrodynamic sizes of ML, FL, and MFL in DMEM with 10% FBS were determined to be ~43, ~337, and ~474 nm, respectively, showing agglomerate consisting of only a few (less than 3) particles. This result corroborated that the particles of nanohybrids can be separated into a smaller assembly in the physiological conditions containing proteins and that the formation of large agglomerates may not affect nanoparticulate properties of nanohybrids negatively, for their cellular uptake. Furthermore, we have verified, in the previous study, that the LDH or drug/LDH nanohybrid was taken up by cells in a small assembly even though they seemed to form agglomerate through TEM study [62]. It was considered that the biological system could sense single particles or small agglomerates of nanoparticles. Comparing with FL and MFL nanohybrids, ML showed much more reduction in hydrodynamic size (Figure 5(a)). It can be explained by the surface interaction between nanohybrids and proteins in the media. The surface of ML and FL nanohybrids was thought to be covered with MTX and 5-FU, respectively, which had different affinity toward proteins; MTX was reported to exhibit strong interaction with albumin [63], while 5-FU had low affinity for albumin [64]. The drug molecules attached on the surface of nanohybrids affected attraction to albumin, resulting in the difference in agglomeration degree. The relatively small hydrodynamic size ~43 nm of ML compared with the lateral particle size ~337 and 474 nm in the SEM (Figure 1(c)) was attributed to the bending of thin particles in the aqueous condition.

**Figure 6:**
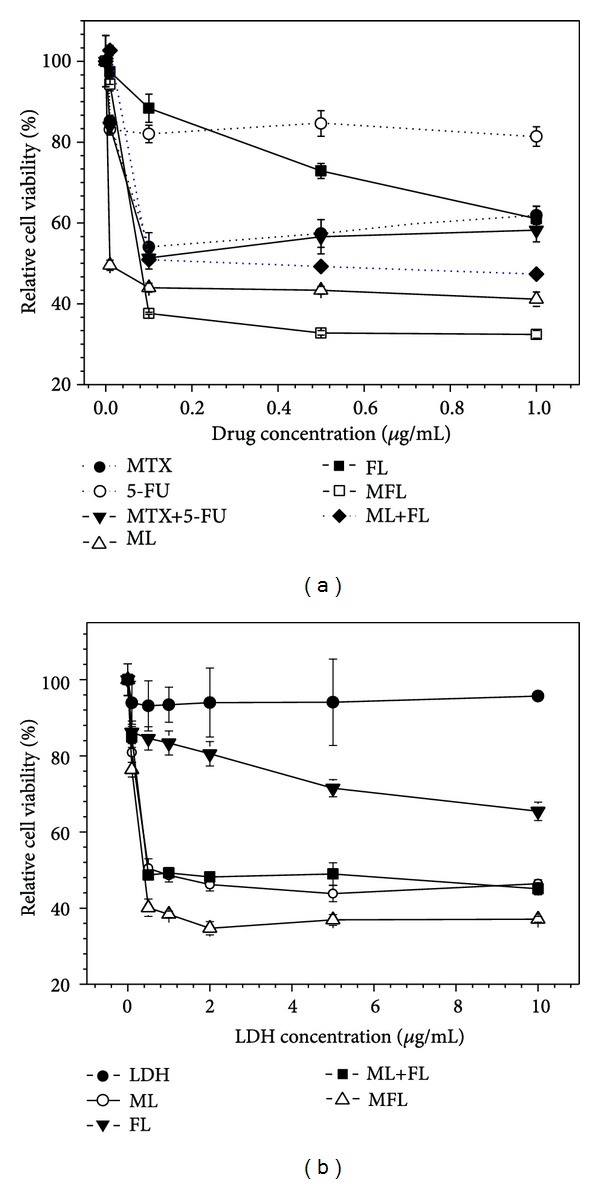
Anticancer efficacy of drug/LDH nanohybrids based on (a) drug concentration and (b) LDH concentration in HeLa cells.

The anticancer therapeutic efficacy of the LDH nanohybrids was investigated by measuring the inhibition of cancer cell proliferation via MTT assay in HeLa cells. The experiments were performed based on drug and LDH (inorganic nanocarrier part) concentration, respectively. When the efficacy of LDH nanohybrids was compared with free drugs or their mixture (Figure 6(a)), ML and FL nanohybrid showed higher anticancer efficacy than each corresponding free drug, which was probably due to the different cellular uptake mechanism of LDH nanohybrids from free drug molecules [2], resulting in the avoidance of drug resistance [30]. In the case of MTX + 5-FU mixture, a similar result was obtained. Anticancer efficacy of MTX was found to be superior to that of 5-FU in HeLa cells and this tendency was also identified in comparison result between ML and FL, which meant higher drug efficacy of MTX than 5-FU as reported previously [4]. Finally, MFL nanohybrid exhibited the highest suppression efficacy on cancer cells in comparison with all the other controls tested. Interestingly, anticancer activity of MFL nanohybrid was more effective than that of ML + FL mixture. This result indicated the importance of codelivery of drug molecules in time and space because different rate of cellular uptake and release site of drugs may hamper the therapeutic effect of combinatorial drug treatment. Considering the minimal cytotoxicity of LDH itself, MFL nanohybrid was proven to possess the greatest potential for cancer therapy by taking advantages of LDH carrier system and combination treatment strategy.

On the other hand, the amounts of LDH in each nanohybrid were not identical because of the difference of the drug contents (wt%) in each nanohybrid. Therefore, anticancer efficacy of LDH nanohybrids was also examined based on treated LDH concentration. In Figure 6(b), MFL nanohybrid showed the highest inhibition efficacy of cancer cells with the following order: MFL > ML ≈ (ML + FL) > FL. This result demonstrates that MFL possesses the most effective anticancer efficacy even in the carrier dose-dependent manner.

The inorganic nanocarrier LDH only was not proven to affect significantly the viability of cancer cells (Figure 6(b)), showing viability higher than 90% at every concentration tested. Therefore, the enhanced therapeutic effect of hybrids compared with drug only was certainly attributed to the drug transportation ability of drug/LDH hybrids. We also evaluated time-dependent drug release pattern from each drug/LDH hybrid in deionized water and DEME media (Figure S2). Drug release was larger in DMEM media than in deionized water, which was attributed to the exchangeable ions in DMEM. The total accumulated drug release at 6 hours was determined to be higher than 50% in DMEM. Therefore we could conclude that the drug/LDH hybrids taken up by cancer cells effectively release loaded drug to intracellular system, resulting in enhanced drug efficacy.

## 4. Conclusion

We have demonstrated the preparation, physicochemical properties, and anticancer efficacy of drug-incorporated LDH nanohybrids. In this work, we adopted reconstruction route to accommodate drug molecules into LDHs preserving topochemical properties of pristine LDHs. Drug molecules such as MTX, 5-FU, and their combination were successfully incorporated to LDH through reconstruction methods preserving the lateral size of LDH nanoparticles. All the nanohybrids were determined to have positive zeta potential in cell culture media with 10% FBS, suggesting the facilitated interaction between nanohybrids and cancer cells. Furthermore, proteins in physiological condition were proven to reduce the degree of agglomerates in nanohybrids, possibly helping them to be recognized by the cancer cells. From the anticancer efficacy assay utilizing human cervical cancer cell, HeLa, we found that the nanohybrids showed higher drug efficacy compared with free drug only. It was notable that the MFL nanohybrid which accommodated MTX and 5-FU together showed the highest anticancer efficacy compared with the combined administration of MTX + 5-FU and ML + FL. These results strongly suggested that the combination anticancer therapy could be enhanced by the co-incorporation of drug cocktail into LDHs.

## Supplementary Material

Although it was difficult to estimate the lateral particle size of drug/LDH nanohybrids due to the randomly stacked thin and bended layers, we found that the lateral sizes of drug/LDH nanohybrids were ~225 ± 14, ~236 ± 12, and ~234 ± 14nm for ML, FL, and MFL, respectively, from repeated SEM measurements

## Figures and Tables

**Table 1:** Chemical formulae and drug contents of drug/LDH nanohybrids.

Sample	Chemical formula	MTX content (wt%)	5-FU content (wt%)	Theoretically maximum drug content (wt%)	
				MTX	5-FU
ML	[Mg_2_Al(OH)_6_][(MTX)_0.19_(OH^−^)_0.62_]1.7H_2_O	28.3	—	~52	—
FL	[Mg_2_Al(OH)_6_][(5-FU)_0.21_(OH^−^)_0.79_]1.7H_2_O	—	10.9	—	~39
MFL	[Mg_2_Al(OH)_6_][(MTX)_0.15_(5-FU)_0.58_(OH^−^)_0.12_]2.0H_2_O	19.0	21.0	N.D.	N.D.
