# Supplementary material for: Anticancer Drug-Incorporated Layered Double Hydroxide Nanohybrids and Their Enhanced Anticancer Therapeutic Efficacy in Combination Cancer Treatment
Source: Biomed Res Int. 2014 Apr 17;2014:193401. doi: 10.1155/2014/193401 (PMC4016841; doi:10.1155/2014/193401)
Supplement: Supplementary file 1 — Although it was difficult to estimate the lateral particle size of drug/LDH nanohybrids due to the randomly stacked thin and bended layers, we found that the lateral sizes of drug/LDH nanohybrids were ~225 ± 14, ~236 ± 12, and ~234 ± 14nm for ML, FL, and MFL, respectively, from repeated SEM measurements [file 193401.f1.pdf]

Supporting information

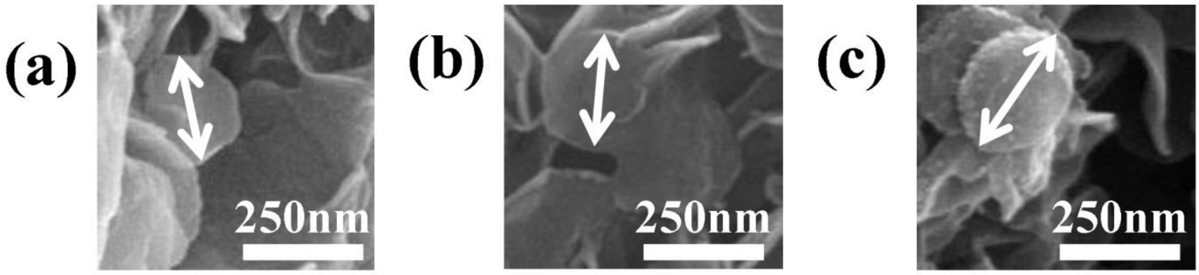

Fig. S1. Scanning electron microscopic images of primary particle of drug/LDH nanohybrid after reconstruction process (a) ML (~ 225 ± 14 nm), (b) FL (~ 236 ± 12 nm) and (c) MFL (~ 234 ± 14 nm)

The lateral particle size of drug/LDH nanohybrid was investigated with the flattened particles. Randomly selected 10 particles from 3 different spots were utilized to obtain average and standard deviation. The lateral sizes of ML, FL and MFL were determined to be ~225±14 nm, ~236±12 nm and~234±14 nm, respectively. Compared to the lateral size of pristine LDH (~233±12) and calcined one (~236±13), those values are statistically considered same according to the student t-test with 95% confidentiality.

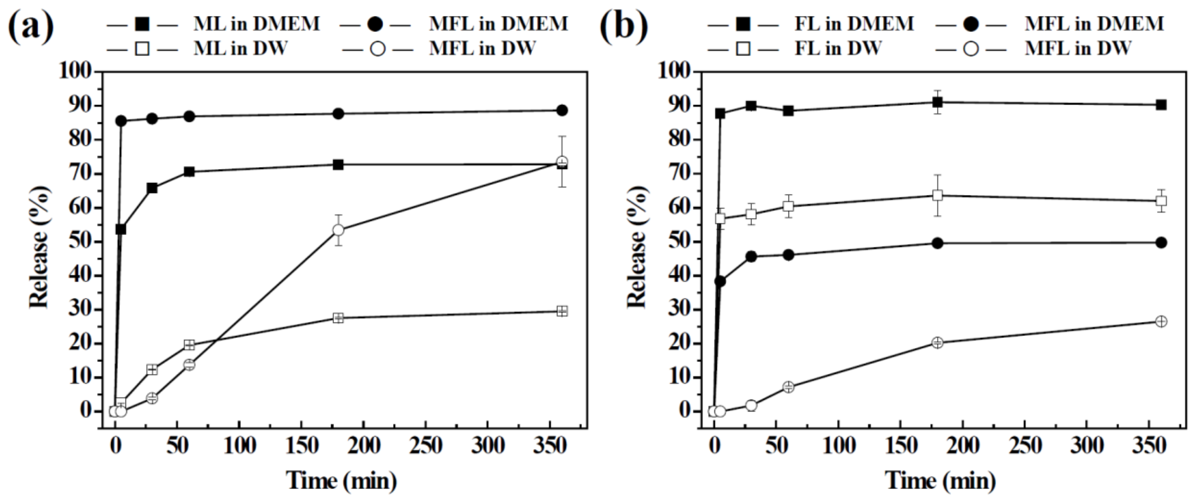

Fig. S2 Time-dependent drug release pattern from ML, FL and MFL hybrids in deionized water (DW) and DMEM cell culture media for (a) MTX (b) 5-FU

Time-dependent drug release pattern from ML, FL and MFL hybrids showed typical L-type curve. Drug release was more accelerated in DMEM compared with DW, which was attributed to the ion exchangeable electrolytes in DMEM media. Accumulated MTX release amounts at 6 h were ~ 70% and ~ 90% in DMEM from ML and MFL, respectively, suggesting that the incorporated drugs could be successfully release from the drug/LDH hybrids. 5-FU also showed accumulated release amounts of ~ 90% and ~ 50% in DMEM from FL and MFL hybrids, respectively, at 6 h. Interestingly, MTX release was more facilitated in MFL compared with ML; FL release was less in MFL than in FL. This might be attributed to the different location of MTX and 5-FU in MFL hybrids arising from different molecular dimension. The larger MTX molecules might be incorporated in the outer part of the hybrid, and thus the release was faster than 5-FU. As most of the drug moiety could be unloaded from the hybrids, the hybrids taken up by cancer cells could effectively transport drug molecules to intracellular system.
